# The association between birth by cesarean section and adolescent cardiorespiratory fitness in a cohort of 339,451 Swedish males

**DOI:** 10.1038/s41598-020-75775-2

**Published:** 2020-10-29

**Authors:** Lucas D. Ekstrom, Viktor H. Ahlqvist, Margareta Persson, Cecilia Magnusson, Daniel Berglind

**Affiliations:** 1 Department of Global Public Health, Karolinska Institutet, Tomtebodavägen 18A, 171 77 Stockholm, Sweden; 2 Department of Nursing, Umeå University, Umeå, Sweden; 3 Centre for Epidemiology and Community Medicine, Region Stockholm, Stockholm, Sweden

**Keywords:** Risk factors, Cardiovascular biology

## Abstract

Birth by cesarean section is increasing worldwide and associates with offspring morbidities capable of adversely impacting cardiorespiratory fitness later in life. Whether birth by cesarean section associates with lower levels of cardiorespiratory fitness later in life is unknown and is of interest to public health. Four Swedish national registers were linked to follow 339,451 singleton males, born between 1973–1987 until December 31 2005, for Watt-maximum achieved on a cycle ergometer test at conscription into the Swedish military. Main exposure was birth by cesarean section which was compared to vaginal birth. A sub-population of 45,999 males born between 1982–1987 was identified to explore differentiated associations between elective and non-elective cesarean section with Watt-maximum. Within-family analyses of 34,252 families with 70,632 biological male siblings, who conscripted during the study period, were performed to explore the role of familial confounding on Watt-maximum. Swedish males born by cesarean section achieved lower mean Watt-maximum (− 2.32 W, 95%C.I. − 2.90 to − 1.75) and displayed excess odds of low cardiorespiratory fitness (aOR = 1.08, 95%C.I. 1.05 to 1.11) at conscription in the eighteenth life-year compared to males born vaginally after adjusting for birth characteristics, maternal morbidities and parental socioeconomic position. In the sub-population, males born 1982–1987, there was a greater negative association of elective cesarean section with cardiorespiratory fitness (− 4.42 W, 95%C.I. − 6.27 to − 2.57, p < 0.001) than non-elective cesarean sections (− 1.96 W, 95%C.I. − 3.77 to − 0.16, p = 0.033) as compared to vaginal births. No associations between modes of cesarean delivery and cardiorespiratory fitness levels persisted in the within-family analyses where biological male siblings were compared whilst controlling for factors shared within families. Males born by cesarean section had lower levels of cardiorespiratory fitness eighteen years later compared to males born vaginally. These findings appear to be largely explained by factors of familial confounding.

## Introduction

The incidence of cesarean section (CS) is increasing worldwide and the global prevalence nearly doubled from 12.1% of all births in 2000 to 21.1% in 2015^1–5^. Maternal and offspring health outcomes associated with CS are consequently of growing interest for public health but are not fully understood. CS rates between 10 and 15% at the population level have been suggested as motivated from a medical viewpoint on the basis that no reductions in maternal or fetal mortality can be found above these rates^6–8^. On the one hand, CS is a vital-indication intervention that reduces maternal and fetal mortality and morbidity. On the other, maternal medical histories of CS are associated with persistent severe obstetric risks in later pregnancies as well as with an excess maternal morbidity burden^9–17^. Epidemiological studies have shown associations between birth by CS with excess risks of a range of offspring morbidities each capable of adversely impacting cardiorespiratory fitness (CRF) later in life^10,18–23^. If CS is a causal factor in such morbidities, it is possible that CS would impact CRF. CRF is a gender-independent predictor of mortality, morbidity and long-term survival^24–26^. Levels of CRF are inversely associated with risks of all-cause mortality, premature death, death from cardiovascular disease and cancer independent of traditional risk factors such as obesity, hypertension, hypercholesterolemia and socioeconomic position^27–33^.The high prognostic value of CRF on long-term survival and disease-specific morbidities throughout an individual’s lifetime underscores the importance to public health of understanding drivers of CRF and targeting early-life interventions to improve CRF. We posited three specific research questions; (i) whether birth by CS in Swedish males born 1973–1987 are associated with lower levels of CRF later in life; (ii) whether such an association differs between elective and non-elective CS in a sub-population of males born 1982–1987 and (iii) whether any associations found robustly persist across within-family analyses.

## Materials and methods

### Study design

We conducted a population-based longitudinal cohort study using nationwide register-linked data on Swedish males born between 1973–1987. The analytic sample was followed until December 31, 2005 for Watt-maximum (Wmax) achieved on a cycle ergometer test during standardized nationwide conscription into the Swedish military. To explore associations of modes of cesarean delivery with Wmax we identified a sub-population of males born between 1982–1987 for which register data allowed differentiation between elective and non-elective CS. To investigate the impact of familial confounding on Wmax we identified biological brothers in the analytic sample and performed within-family sibling comparison analyses.

### Data sources

The unique Swedish personal identity number was used to link four Swedish nationwide registers^34^. The Swedish Medical Birth Register (MBR), containing compulsory reported data from all birth clinics on nearly all deliveries in Sweden since 1973, was used to collect data on modes of delivery, birth characteristics and maternal medical histories^35^. The Swedish Military Service Conscription Register was used to collect CRF data at conscription into the Swedish military. The Population and Housing Censuses (PHC) were used to collect information on parental education levels, household disposable income, parental country of birth and parental socioeconomic position. The Swedish Multigeneration Register facilitated linking of individual family members into family clusters permitting within-family analyses of biological male siblings.

### Study population

**Figure 1:**
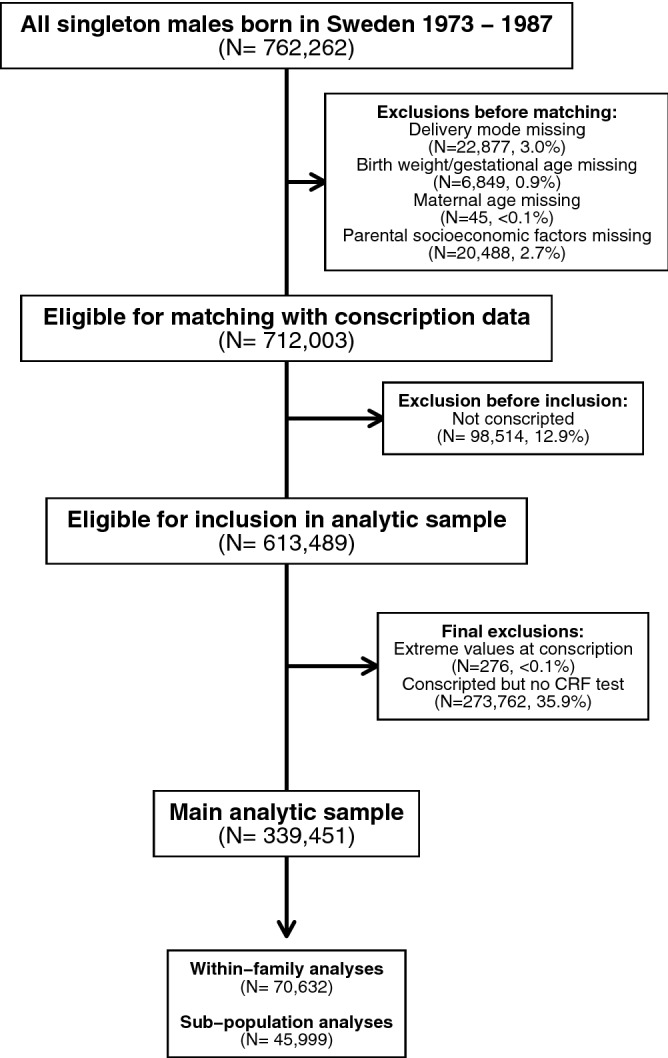
Construction of main analytic sample, family clusters and analytic sub-population.

A flow diagram detailing the construction of the analytic sample, family clusters and analytic sub-population and are shown in Fig. 1. The study population of all Swedish singleton males born between 1973–1987 (N = 762,262) was identified from the MBR. Individuals where data was missing on delivery mode (N = 22,877, 3.0%), birth weight and/or gestational age (N = 6849, 0.9%), maternal age (N = 45, < 0.1%) and parental socioeconomic variables (N = 20,488, 2.7%) were excluded. Further, individuals who did not conscript into Swedish military service during the study period (N = 98,514, 12.9%) were excluded. The resulting cohort (N = 613,489, 80.5%) was matched with conscription data as recorded in the Swedish Military Service Conscription Register. Individuals with extreme values of height (below 150 cm or above 210 cm), weight (below 40 kg or above 150 kg) or body-mass index (BMI) (below 15 kg/m^2^ or above 60 kg/m^2^) (N = 276, < 0.1%) as well as individuals who conscripted but were not allowed to perform the maximum load cycle ergometer test (N = 273,762, 35.9%) were excluded to define the main analytic sample (N = 339,451). To explore the role of familial confounding on Wmax, we identified 34,252 families with 70,632 biological brothers in the main analytic sample who all conscripted during the study period. To explore the associations of modes of cesarean delivery with Wmax, we defined a sub-population of males born 1982–1987 (N = 45,999) where MBR data allowed differentiation between elective and non-elective CS deliveries, respectively.

### Exposure and controls

Exposure in the main analytic sample was defined as births by CS (N = 29,910, 8.8%) and these were compared to vaginal births (N = 309,541, 91.2%). In the analytic sub-population of males born 1982–1987, exposures were defined as elective (N = 1903, 4.1%) and non-elective (N = 1958, 4.3%) CS, respectively and compared to vaginal births (N = 42,138, 91.6%). The reporting standards of the MBR remained constant throughout the study period and defined elective CS as CS delivery before the onset of labor, and non-elective CS as CS delivery after the onset of labor.

### Outcome

Wmax, a validated proxy measure of CRF^36^, was defined as outcome. During the study period conscription in Sweden was mandated by law and exemption from conscription required stated medical approval, usually granted to those with severe medical conditions. All recruits called for conscription in the eighteenth life-year, who were considered medically fit, were invited to take the adjustable resistance cycle ergometer test during standardized nationwide conscription. Individuals eligible for conscription but who were not considered medically fit during conscription testing due to muscular och skeletal injuries, abnormal resting ECG or other relevant medical indications were excluded from performing the ergometric testing by supervising physicians. The maximum work output achieved by each recruit at exhaustion, measured in Watts, was recorded as their respective Wmax. We quantified associations between modes of delivery with mean Wmax and with low CRF, where we ordered Wmax into quartiles and categorically defined low CRF as the lowest quartile of recruits (≤ 270 Wmax). Within-family analyses in the linear regression model were performed on 70,632 continuously outcome discordant siblings and on 20,590 categorically outcome discordant siblings in the logistic model.

### Other covariates

We used known indications for cesarean delivery and predictors of fetal health and development as covariates in our main analyses. We adjusted for birthweight and gestational age since both are positive predictors of CRF later in life^37,38^. Maternal covariates at the time of delivery included age^39^, parity^40^, diabetes^41,42^, hypertension^43^, preeclampsia during pregnancy^44,45^ and systemic lupus erythematosus^46,47^. Increased data coverage in the MBR after 1982 allowed additional adjustments in our analyses on the sub-population of males born 1982–1987 of pre-pregnancy BMI^40,48^ and self-reported smoking habits during pregnancy^49,50^. From the PHC we acquired data collected from the census conducted closest to the time of delivery on highest parental educational level, household disposable income (in quintiles), parental country of birth (Sweden or one/two parents born outside Sweden) and highest parental occupational class.

### Statistical analysis

Linear and logistic regression were employed to estimate mean difference in Wmax and odds ratios (OR) of low CRF in males born 1973–1987 by any CS as compared to males born vaginally. The same methodology was applied to males in the analytic sub-population born 1982–1987, where we differentiate between elective and non-elective CS as compared to males born vaginally. Within-family analyses employed fixed-effects (conditional) linear and logistic regressions, thereby accounting for genetic and environmental familial factors shared between biological brothers. Within-family analyses were adjusted similarly as other analyses, with the exception of parental education and parental country of birth which did not vary between brothers. For all analyses standard errors were estimated using the robust (sandwich) method to account for correlation between biological brothers. Significance tests were two sided and statistical analyses were performed using STATA 15.1 (Stata Corp.)

### Sensitivity analysis

To explore possible heterogeneity in vaginal births we differentiated vaginal delivery with forceps and/or vacuum extraction from non-instrumental vaginal delivery, Supplementary Table S2. To verify that individuals excluded because of extreme-value conscription data did not materially impact our findings, we included those previously excluded (Model 1). To relax linearity assumptions of covariates, we employed restricted cubic splines with five knots at percentiles suggested by Harrell^51^ (5th, 27.5th, 50th, 72.5th and 95th percentiles) for maternal age, household disposable income, gestational age and birthweight standardized according to gestational age (Model 2). To explore the possible influence of both a previous CS and maternal weight gain during pregnancy in the analytic sub-population we adjusted for a maternal medical history of CS after 1972 (Model 3) and standardized maternal gestational weight gain, according to a Swedish reference methodology^52^ (Model 4). To explore the possibility that gestational age is a collider on causal pathways between CS and Wmax we excluded adjustment for gestational age (Model 5). To ascertain that the effect of CS on Wmax is not limited to the extremes of gestational age we excluded pre-term births (< 37 weeks, Model 6) and restricted the analytic sample to males born at-term (Model 7).

### Ethics declaration

The study was approved by the Stockholm ethical review board (Dnr:2016/1445-31/1) and carried out in accordance with relevant guidelines and regulations. Informed consent was not required for analysis of anonymized register data and formally waived by the approving committee. Additional approval from the National Board of Health and Welfare, the Swedish Military Service Conscription Registry and Statistics Sweden was secured prior to collecting the data. All data was collected in accordance with Swedish data privacy legislation and was anonymized using a conversion key held by Statistics Sweden.

## Results

### Population characteristics

Vaginally born males achieved higher Wmax (304.0 W) compared to males born by CS (301.0 W) and were of the same median age during conscription testing (18.3 years old), Table 1. Vaginally delivered males had higher average birth weights (3602.2 g) and longer gestational periods (39.7 weeks) compared to males delivered through CS (3398.8 g, 38.8 weeks), Table 1. Mothers who gave birth through CS were older (28.8 years old), more likely to have a university degree (40.0%) and be primiparas (median parity 1.0) and were part of households with higher disposable income (51.1% quintiles 4–5) compared to mothers who delivered vaginally (27.2 years old, 37.6% university degree, median parity 2.0 and 47.8% quintiles 4–5), Table 1.

**Table 1:** Population characteristics main analytic sample (males born 1973–1987) by exposure category.

Outcome and covariates	Males born 1973–1987 N = 339,451 (100.0%)	Vaginal delivery^a^ N = 309,541 (91.2%)	Cesarean section^a,b^ N = 29,910 (8.8%)
Wmax (W), mean (SD)	303.7 (49.0)	304.0 (49.1)	301.0 (48.5)
Low CRF (lowest quartile), number (%)	86,464 (25.5%)	78,389 (25.3%)	8075 (27.0%)
Age at conscription (years), median (IQR)	18.3 (18.1, 18.5)	18.3 (18.1, 18.5)	18.3 (18.1, 18.5)
Birth weight (g), mean (SD)	3584.3 (530.9)	3602.2 (514.0)	3398.8 (653.6)
Weeks of gestation (weeks), mean (SD)	39.7 (1.8)	39.7 (1.7)	38.8 (2.2)
Maternal age at birth (years), mean (SD)	27.4 (4.9)	27.2 (4.8)	28.8 (5.5)
Parity (number) median (IQR = Q3 – Q1)	2.0 (1.0, 2.0)	2.0 (1.0, 2.0)	1.0 (1.0, 2.0)
**Maternal diseases at time of giving birth**			
Diabetes, number (%)	1233 (0.4%)	708 (0.2%)	525 (1.8%)
Hypertension, number (%)	262 (0.1%)	203 (0.1%)	59 (0.2%)
Preeclampsia, number (%)	1459 (0.4%)	1045 (0.3%)	414 (1.4%)
Systemic lupus erythematosus, number (%)	29 (< 1%)	22 (< 1%)	7 (< 1%)
**Highest parental educational level**			
Primary education, number (%)	43,816 (12.9%)	39,916 (12.9%)	3900 (13.0%)
Secondary education, number (%)	167,390 (49.3%)	153,358 (49.5%)	14,032 (46.9%)
University degree or higher, number (%)	128,245 (37.8%)	116,267 (37.6%)	11,978 (40.0%)
**Household disposable income (quintiles 1–5)**			
Quintile 1	27,715 (8.2%)	25,057 (8.1%)	2658 (8.9%)
Quintile 2	63,044 (18.6%)	57,826 (18.7%)	5218 (17.4%)
Quintile 3	85,334 (25.1%)	78,589 (25.4%)	6745 (22.6%)
Quintile 4	83,394 (24.6%)	76,145 (24.6%)	7249 (24.2%)
Quintile 5	79,964 (23.6%)	71,924 (23.2%)	8040 (26.9%)
**Parental country of birth**			
Both parents born in Sweden	299,243 (88.2%)	273,185 (88.3%)	26,058 (87.1%)
One parent born in Sweden	29,445 (8.7%)	26,629 (8.6%)	2816 (9.4%)
Neither parent born in Sweden	10,763 (3.2%)	9727 (3.1%)	1036 (3.5%)
**Highest parental occupational class in childhood**			
Self-employed, farmers and non-categorized	30,222 (8.9%)	27,381 (8.8%)	2841 (9.5%)
Unskilled workers	59,778 (17.6%)	54,899 (17.7%)	4879 (16.3%)
Skilled workers	65,380 (19.3%)	60,119 (19.4%)	5261 (17.6%)
Non-manual workers (lower level)	51,617 (15.2%)	47,073 (15.2%)	4544 (15.2%)
Non-manual workers (intermediate level)	86,444 (25.5%)	78,770 (25.4%)	7674 (25.7%)
Non-manual workers (higher level)	46,010 (13.6%)	41,299 (13.3%)	4711 (15.8%)

^a^Exposure in the main analytic sample was defined as males born by cesarean section (elective and non-elective undifferentiated) and were compared to males born vaginally.

^b^Cesarean section shows non-differentiated CS (elective and non-elective CS combined).

Population characteristics of the analytic sub-population of males born 1982–87 were similar compared to the main analytic sample, Supplementary Table S1. Mothers who gave birth through non-elective CS had a higher pre-pregnancy BMI (17.7% overweight or obese) and were more likely to smoke during pregnancy (29.9% smokers) compared to mothers who delivered vaginally (12.6% overweight or obese, 25.5% smokers) and mothers who delivered through elective CS (16.6% overweight or obese, 25.9% smokers), respectively, Supplementary Table S1. Mothers who gave birth through elective CS were part of households with higher disposable income (39.7% quintiles 4–5) compared to mothers who delivered vaginally and through non-elective CS (33.2% and 33.9% quintiles 4–5 respectively). Population characteristics of excluded individuals are shown in Supplementary Table S4.

### Cesarean delivery and cardiorespiratory fitness

**Figure 2:**
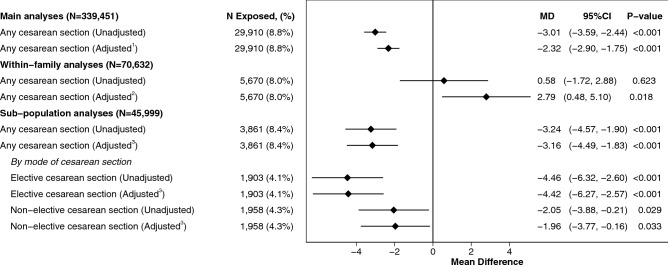
Association between cesarean section and Wmax performance at conscription in the 18th life-year for the main analytic sample, within-family analyses and analytic sub-population. Superscript 1: Adjusted models in main analyses show linear regression estimates of mean difference in Wmax, as measured in Watts (W), 95% confidence intervals and p-values between individuals born through cesarean section (elective and non-elective combined) as compared to all individuals born vaginally. Adjustments included: birthweights standardized according to gestational age, gestational age, maternal age, parity, maternal diseases (diabetes, hypertension, preeclampsia, SLE), highest parental educational level, household disposable income, parental country of birth and highest parental occupational class. Superscript 2: Within-family analyses were similar in terms of adjustments (excluding parental country of birth and highest parental education) to the main analyses and were performed on all biological siblings in the main analytic sample who conscripted during the study period. Superscript 3: Adjusted models in the analytic sub-population show linear regression estimates of mean difference in Wmax, as measured in Watts (W), 95% confidence intervals and p-values between individuals born through cesarean section (elective and non-elective combined as well as differentiated by mode of cesarean delivery) as compared to all individuals born vaginally. Adjustments included: birthweights standardized according to gestational age, gestational age, maternal age, parity, maternal diseases at time of giving birth (diabetes, hypertension, preeclampsia, SLE) and parental educational level, disposable income, country of birth and occupational class as well as pre-pregnancy BMI and self-reported maternal smoking habits during pregnancy.

**Figure 3:**
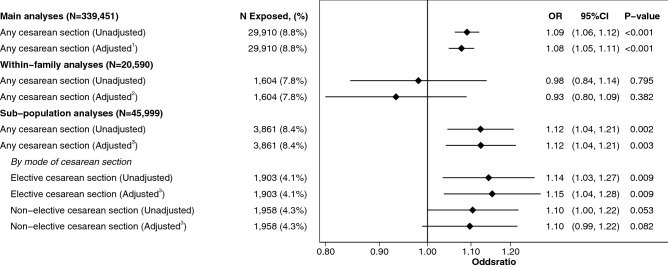
Association between cesarean section and low cardiorespiratory fitness at conscription in the 18th life-year for the main analytic sample, within-family analyses and analytic sub-population. Superscript 1: Adjusted models in main analyses show logistic regression estimates of odds ratios of low CRF (defined as lowest quartile of Wmax achieved by all recruits during the study period), 95% confidence intervals and p-values between individuals born through cesarean section (elective and non-elective combined) as compared to all individuals born vaginally. Adjustments included: birthweights standardized according to gestational age, gestational age, maternal age, parity, maternal diseases (diabetes, hypertension, preeclampsia, SLE), highest parental educational level, household disposable income, parental country of birth and highest parental occupational class. Superscript 2: Within-family analyses were similar in terms of adjustments (excluding parental country of birth and highest parental education) to the main analyses and were performed on all biological siblings in the main analytic sample who conscripted during the study period. Superscript 3: Adjusted models in sub-population show logistic regression estimates of odds ratios of low CRF (defined as lowest quartile of Wmax achieved by all recruits during the study period), 95% confidence intervals and p-values between individuals born through cesarean section (elective and non-elective combined as well as differentiated by mode of cesarean delivery) as compared to all individuals born vaginally. Adjustments included: birthweights standardized according to gestational age, gestational age, maternal age, parity, maternal diseases at time of giving birth (diabetes, hypertension, preeclampsia, SLE) and parental educational level, disposable income, country of birth and occupational class as well as pre-pregnancy BMI and self-reported maternal smoking habits during pregnancy.

Male offspring born by CS between 1973–1987 achieved lower Wmax (− 2.32 W, 95%C.I. − 2.90 to − 1.75, p < 0.001, Fig. 2) and showed excess odds of low CRF (aOR = 1.08, 95%CI 1.05 to 1.11, p < 0.001, Fig. 3) compared to males born vaginally after adjusting for birth characteristics, maternal morbidities and socioeconomic position. When comparing 70,632 biological siblings across 34,252 families in the main analytic sample, of which 4750 and 1479 siblings were fully exposure and outcome discordant in the linear (Fig. 2) and logistic (Fig. 3) model respectively, the associations of CS with Wmax at conscription attenuated towards the null, with the exception of birth by any CS (elective and non-elective combined) in the within-family linear regression model which showed a statistically significant positive association with Wmax after fully adjusting for covariates (2.79 W, 95%C.I. 0.48 to 5.10, p = 0.018, Fig. 2). Associations with Wmax did not differ between males born by instrumental vaginal birth and those delivered by unassisted vaginal births suggesting no impact on Wmax from heterogeneity in vaginal births, Supplementary Table S2. Associations between birth by CS with lower levels of CRF, as compared to vaginal births, were stable and persisted across all sensitivity analyses performed, Supplementary Table S3.

### Modes of cesarean delivery and cardiorespiratory fitness

For males in the analytic sub-population born 1982–1987 associations of birth by any cesarean delivery method with Wmax were similar to the main analytic sample after similar adjustments with the addition of pre-pregnancy BMI and self-reported maternal smoking habits during pregnancy, Fig. 2. When differentiating between modes of cesarean delivery and after adjusting for confounders, both elective and non-elective CS associated with lower mean Wmax at conscription (Mean difference = − 4.42, 95%CI − 6.27 to − 2.57, p < 0.001 and − 1.96, 95%CI − 3.77 to − 0.16, p = 0.033 respectively, Fig. 2) as compared to vaginal delivery. Elective CS in the logistic model associated with increased odds of low CRF at conscription (aOR = 1.15, 95%CI 1.04 to 1.28, p = 0.009, Fig. 3). Non-elective CS associated with increased odds of low CRF at conscription (aOR = 1.10, 95%CI 0.99 to 1.22, Fig. 3) although not statistically different from vaginal deliveries (p = 0.082). There was no difference between elective CS and non-elective CS in their association to mean Wmax (p = 0.055) and odds of low CRF (p = 0.505), as compared to those born by vaginal delivery.

## Discussion

### Principal findings

Our analysis of data from Swedish nationwide health and administrative registers, of 339,451 singleton males born 1973–1987, suggested that males born by CS have lower levels of CRF in the eighteenth life-year compared to males born vaginally. The association between CS with low CRF persisted across all sensitivity analyses performed. Analysis of data in our sub-population of 45,999 singleton males born 1982–1987, for which modes of cesarean delivery were differentiated, indicated that both elective and non-elective cesarean delivery associated with lower levels of CRF later in life as compared to vaginal delivery, albeit not statistically significant in the case of non-elective CS. When comparing 70,632 biological siblings across 34,252 families who all conscripted during the study period, the negative association between exposure to birth by any CS with levels of CRF later in life initially found in our main analysis inverted to a statistically significant positive association after fully adjusting for covariates.

### Interpretation of findings

Our main analysis captured a modest but statistically significant association between birth by CS and lower levels of CRF. However, this negative association between CS with levels of CRF later in life inverted when accounting for genetic and environmental factors and our linear regression within-family model estimated a positive association between birth by any CS and Wmax after fully adjusting for covariates. Rather than overreaching in our interpretation of the relevance of this positive association, which we consider more sporadic in nature than clinically relevant, we interpret the findings of our within-family analyses to indicate a full attenuation of the negative association between birth by CS with lower levels of CRF later in life as initially indicated in our main analysis.

Thus, residual confounding capable of explaining the negative association found in our main analysis is relevant to consider in this context. Firstly, it is known that physical activity (PA) in adult populations in high Human Development Index (HDI) countries is positively associated with socioeconomic position, with a stronger impact in the first half of one’s lifetime^53–56^. In turn, low to moderate physical exercise during pregnancy, particularly in the second and third trimester, has been shown to reduce the risk for cesarean delivery^57,58^. In addition, maternal obesity is a known indication of CS in general and elective CS in particular, and is strongly inversely associated with both socioeconomic position and PA, particularly in high-HDI countries^59,60^. That is to say, although we controlled for socioeconomic position through a range of relevant covariates it is possible that our main analyses are still impacted by unmeasured residual socioeconomic confounding. Secondly, another possibility is that shared genetic predisposition and behavioral modulators of CRF phenotype in our within-family analysis accounts for the attenuation observed. CRF phenotype is a strong gender-independent predictor of individual mortality and morbidity independent of PA-levels^61^. Although CRF is subject to genetic predisposition, it is also clearly subject to modulation through behavioral attitudes towards PA participation and physiological factors^62^. By thus comparing biological siblings in our within-family analysis the shared genetic predisposition for CRF phenotype as well as the shared behavioral and physiological modulating factors of CRF could thus all contribute towards the attenuation. If genetic predisposition for CRF phenotype would also have a bearing on the probability of a CS delivery, for example via maternal obesity^63^, it could be material in explaining our within-family attenuation. E-values for our estimates indicate that an unmeasured confounder that increased the odds of the outcome by 26% in either the exposed or unexposed group, and if that unmeasured confounder was 26% more prevalent among the exposed than the unexposed, would suffice to fully attenuate the association^64,65^. Altogether, analysis of the available data on balance does not support the likelihood of a causal link between birth by CS and low levels of CRF later in life. Instead it favors an interpretation where residual confounding, possibly socioeconomic or genetic in nature, likely accounts for the initially observed associations that attenuate across within-family analyses where individuals exposed to the same socioeconomic and genetic modulators of CRF are compared.

### Clinical relevance

Rapidly increasing global incidence of CS makes systematic investigation of offspring health outcomes associated with birth by CS a growing area of interest for public health. Of particular importance is exploration of associations between CS and gender-independent predictors of long-term survival and all-cause mortality suitable for intervention. Besides genetic predisposition for disease-specific morbidities, CRF is perhaps one of the stronger known predictors of long-term survival that it lends itself to policy-driven intervention at the population-level to improve public health outcomes^24–30,66^. We used Wmax as a validated proxy outcome for CRF and did not find any associations between birth by CS and CRF in the eighteenth life-year that was not completely explained by factors of familial confounding.

Wmax has been repeatedly validated in the literature as a close correlate of maximal oxygen uptake (VO_2_ max) and is considered a relevant proxy for measuring CRF^67–69^. Wmax can be used to estimate VO_2_ max using formulaic methodology that allows for CRF comparisons across VO_2_ max which is the global standard of CRF^36^. In our study we defined low CRF as the lowest quartile of recruits in terms of Wmax achieved, and this translated to a Wmax cut-off value of 270 W. Converting our low CRF cut-off value into VO_2_ max for a young adolescent weighing 75 kg would correspond to around 42.3 mL/kg/min and is in-line with the 42 mL/kg/min VO_2_ max cut-offs proposed in the literature for identifying adolescents with increased risk of cardiovascular disease^70^.

### Strengths and limitations

The use of a validated and objectively measured proxy for CRF collected in a standardized test environment, access to high-quality national registers with broad data ranges and total population coverage, the large sample size and long-term follow-up are major strengths of this study. The ability to differentiate between elective and non-elective CS in a large sub-population of males born 1982–1987 is a medically relevant strength as both indications and risk-profiles differ and thus control for a potential source of confounding by indication. Adjustments of a wide range of possible socioeconomic confounders in conjunction with our within-family analysis that likely captures additional unmeasured residual socioeconomic confounding contribute to the robustness of our conclusions.

Yet, residual confounding can never be excluded in an observational study and there are limitations that must be considered. Firstly, our analytic sample contains only males due to sex-biased conscription legislation in Sweden during the study period. Secondly, although within-family analyses are robust to unobserved shared familial confounding they are still sensitive to non-shared confounding^71^, shared-mediators^72^, and measurement error^71^. While we believe measurement errors in our outcome are minimal due to the standardized nature of data collection and even though we controlled for non-shared observed confounding, caution may still be warranted when directly interpreting coefficients from sibling analyses. Thirdly, our findings are potentially limited by exclusion bias. Notwithstanding those excluded because of incomplete or missing data at birth, a total of 372,552 males were excluded because they were either not called upon for conscription or were not allowed to perform the CRF test for various medical reasons. Among those excluded we observe a significantly higher proportion of CS births (11.3%) compared to those who conscripted (9.8%) and our main analytic sample (8.8%) as well as a clear trend towards lower socioeconomic position, particularly in those not invited for conscription, Supplementary Table S4. As a result, our final analytic sample covers only about 44.5% of the total male population born between 1973–1987 and must be considered as significantly healthier and possibly hailing from higher socioeconomic positions compared to the general population. That is to say that while our findings are representative for a large population of healthy Swedish males it is likely that those excluded from our study somehow differ systematically which may lead to an underestimation of a potential causal effect of CS on CRF later in life. Lastly, our data coverage did not include PA-data on recruits which meant that we were unable investigate a possible role of PA in the relationship between CS and CRF.

## Conclusions

Our analysis captured modest but statistically significant population-level associations between exposure to birth by CS and lower levels of CRF in the eighteenth life-year. However, these associations attenuated fully in our within-family analyses which take into account factors of familial confounding such as genetic and socioeconomic modulators of CRF. Further research into the possible impact of birth by CS on CRF later in life, particularly in the female population, is warranted and could be of value to public health.

## Supplementary information

Supplementary Information.

## Data Availability

Due to Swedish legal restrictions and the existing ethical approval for this study, data will not be made publically available. The research group can provide descriptive data in table form.

## Abbreviations

CS: Cesarean section
CRF: Cardiorespiratory fitness
Wmax: Watt-maximum
MBR: The Swedish medical birth register
PHC: The population and housing censuses
BMI: Body-mass index
aOR: Adjusted odds ratio
CI: Confidence interval
PA: Physical activity
HDI: Human development index
VO_2_ max: Maximal oxygen uptake
