# Supplementary material for: The association between birth by cesarean section and adolescent cardiorespiratory fitness in a cohort of 339,451 Swedish males
Source: Sci Rep. 2020 Oct 29;10:18661. doi: 10.1038/s41598-020-75775-2 (PMC7596509; doi:10.1038/s41598-020-75775-2)
Supplement: Supplementary file 1 — Supplementary Information. [file 41598_2020_75775_MOESM1_ESM.pdf]

The association between birth by cesarean section and adolescent cardiorespiratory fitness in a cohort of 339,451 Swedish males

Lucas D. EKSTROM[1*] (0000-0002-0377-6002)

Viktor H. AHLQVIST[1] (0000-0003-1383-3194)

Margareta PERSSON[2] (0000-0002-5300-0990)

Cecilia MAGNUSSON[1,3] (0000-0002-8567-6725)

Daniel BERGLIND[1,3] (0000-0003-0616-7779)

[1]Department of Global Public Health, Karolinska Institutet, Stockholm, Sweden

[2]Department of Nursing, Umeå University, Umeå, Sweden

[3]Centre for Epidemiology and Community Medicine, Region Stockholm, Sweden

***Correspondence to:** Lucas D. EKSTROM, lucas.ekstrom@ki.se Department of Global Public Health, Karolinska Institutet, Tomtebodavägen 18A, 171 77 Stockholm, Sweden.

Population characteristics analytic sub-population (males born 1982-1987) by exposure category

Supplementary Table S1

Outcome and Covariates	Males born 1982-87 N=45,999 (100.0%)	Vaginal delivery[1] N=42,138 (91.6%)	Elective cesarean section[1,2] N=1,903 (4.1%)	Non-elective cesarean section[2] N=1,958 (4.3%)
**Wmax (W), mean (SD)**	296.1 (41.2)	296.4 (41.3)	291.9 (40.4)	294.3 (40.5)
**Low CRF (lowest quartile), number (%)**	12,358 (26.9%)	11,238 (26.7%)	559 (29.4%)	561 (28.7%)
**Age at conscription (years), median (IQR)**	18.3 (18.1, 18.4)	18.3 (18.1, 18.4)	18.3 (18.1, 18.4)	18.3 (18.1, 18.4)
**Birth weight (g), mean (SD)**	3,634.7 (514.7)	3,650.9 (496.9)	3,458.1 (549.6)	3,458.3 (741.0)
**Weeks of gestation (weeks), mean (SD)**	39.5 (1.6)	39.6 (1.5)	38.2 (1.3)	39.1 (2.5)
**Maternal age at birth (years), mean (SD)**	28.5 (4.9)	28.4 (4.8)	30.7 (5.3)	28.7 (5.1)
**Parity (number) median (IQR = Q3 – Q1)**	2.0 (1.0, 2.0)	2.0 (1.0, 2.0)	2.0 (1.0, 3.0)	1.0 (1.0, 2.0)
**Maternal diseases at time of giving birth**				
Diabetes, number (%)	195 (0.4%)	146 (0.3%)	32 (1.7%)	17 (0.9%)
Hypertension, number (%)	93 (0.2%)	74 (0.2%)	7 (0.4%)	12 (0.6%)
Preeclampsia, number (%)	694 (1.5%)	533 (1.3%)	47 (2.5%)	114 (5.8%)
Systemic lupus erythematosus, number (%)	4 (<0.1%)	3 (<0.1%)	0 (0.0%)	1 (0.1%)
**Maternal pre-pregnancy BMI, mean (SD)**	21.9 (2.9)	21.9 (2.9)	22.3 (3.3)	22.4 (3.1)
Underweight (BMI < 18.5), number (%)	3,223 (7.0%)	2,951 (7.0%)	137 (7.2%)	135 (6.9%)
Normal weight (18.5 < BMI < 25), number (%)	36,838 (80.1%)	33,911 (80.5%)	1,450 (76.2%)	1,477 (75.4%)
Overweight (25 < BMI < 30), number (%)	5,129 (11.2%)	4,574 (10.9%)	258 (13.6%)	297 (15.2%)
Obese (25 < BMI < 30), number (%)	809 (1.8%)	702 (1.7%)	58 (3.0%)	49 (2.5%)
**Maternal smoking habits during pregnancy**				
Nonsmoker, number (%)	34,177 (74.3%)	31,394 (74.5%)	1,411 (74.1%)	1,372 (70.1%)
< 10 cigarettes/day, number (%)	7,661 (16.7%)	6,942 (16.5%)	320 (16.8%)	399 (20.4%)
≥ 10 cigarettes/day, number (%)	4,161 (9.0%)	3,802 (9.0%)	172 (9.0%)	187 (9.6%)
**Highest parental educational level**				
Primary education, number (%)	3,555 (7.7%)	3,236 (7.7%)	155 (8.1%)	164 (8.4%)
Secondary education, number (%)	22,562 (49.0%)	20,760 (49.3%)	873 (45.9%)	929 (47.4%)
University degree or higher, number (%)	19,882 (43.2%)	18,142 (43.1%)	875 (46.0%)	865 (44.2%)
**Household disposable income (quintiles 1-5)**				
Quintile 1	9,263 (20.1%)	8,502 (20.2%)	335 (17.6%)	426 (21.8%)
Quintile 2	11,333 (24.6%)	10,465 (24.8%)	413 (21.7%)	455 (23.2%)
Quintile 3	9,994 (21.7%)	9,181 (21.8%)	400 (21.0%)	413 (21.1%)
Quintile 4	8,628 (18.8%)	7,894 (18.7%)	374 (19.7%)	360 (18.4%)
Quintile 5	6,781 (14.7%)	6,096 (14.5%)	381 (20.0%)	304 (15.5%)
**Parental country of birth**				
Both parents born in Sweden	40,768 (88.6%)	37,383 (88.7%)	1,661 (87.3%)	1,724 (88.0%)
One parent born in Sweden	3,895 (8.5%)	3,545 (8.4%)	182 (9.6%)	168 (8.6%)
Neither parent born in Sweden	1,336 (2.9%)	1,210 (2.9%)	60 (3.2%)	66 (3.4%)
**Highest parental occupational class in childhood**				
Self-employed, farmers and non-categorized	4,312 (9.4%)	3,946 (9.4%)	188 (9.9%)	178 (9.1%)
Unskilled workers	6,218 (13.5%)	5,721 (13.6%)	242 (12.7%)	255 (13.0%)
Skilled workers	8,324 (18.1%)	7,666 (18.2%)	309 (16.2%)	349 (17.8%)
Non-manual workers (lower level)	6,781 (14.7%)	6,183 (14.7%)	283 (14.9%)	315 (16.1%)
Non-manual workers (intermediate level)	12,272 (26.7%)	11,251 (26.7%)	510 (26.8%)	511 (26.1%)
Non-manual workers (higher level)	8,092 (17.6%)	7,371 (17.5%)	371 (19.5%)	350 (17.9%)

[1]Vaginal delivery served as the reference for all analyses performed on the analytic sub-population of males born 1982-87
[2]MBR reporting standards remained constant during the study period and defined elective CS as CS delivery before the onset of labor, and non-elective CS as CS delivery after the onset of labor.

Sensitivity Analyses: Adjusted odds ratios, mean differences, 95% confidence intervals and p-values for associations of modes of delivery at birth with Wmax performance at conscription in the eighteenth life-year for all model variations used in sensitivity analyses.

		Logistic regression			*Linear regression*		
**Supplementary Table S2**	**N exposed (%)**	**Odds ratio (low CRF)**	**95% CI**	***p-value***	**Mean difference (Wmax)**	**95% CI**	***p-value***
**Vaginal non-instrumental (Ref.)**	287,431 (84.7%)	**1.00**	-	-	**0**	-	-
Vaginal instrumental (forceps and vacuum extraction) [*]	22,110 (6.5%)	1.02	0.99 to 1.05	0.251	-1.05	-1.72 to -0.38	0.002
Cesarean section[*]	29,910 (8.8%)	1.08	1.05 to 1.11	<0.001	-2.41	-2.99 to -1.83	<0.001

**Supplementary Table S3**	**N total**	**Odds ratio (low CRF)**	**95% CI**	***p-value***	**Mean difference (Wmax)**	**95% CI**	***p-value***
**Cesarean section: Model 1**[1]	339,531	1.08	1.05 to 1.11	<0.001	-2.32	-2.90 to -1.75	<0.001
**Cesarean section: Model 2**[2]	339,451	1.07	1.04 to 1.11	<0.001	-2.01	-2.59 to -1.44	<0.001
**Cesarean section: Model 3**[3]	339,451	1.09	1.06 to 1.12	<0.001	-2.37	-2.96 to -1.78	<0.001
**Cesarean section: Model 4**[4]	45,439	1.13	1.05 to 1.23	0.001	-3.09	-4.43 to -1.75	<0.001
**Cesarean section: Model 5**[5]	339,451	1.10	1.07 to 1.13	<0.001	-2.83	-3.40 to -2.26	<0.001
**Cesarean section: Model 6**[6]	324,552	1.09	1.06 to 1.12	<0.001	-2.67	-3.27 to -2.07	<0.001
**Cesarean section: Model 7**[7]	316,170	1.09	1.06 to 1.13	<0.001	-2.68	-3.29 to -2.06	<0.001

[*]Vaginal instrumental and cesarean section compared all males in the main analytic sample born vaginally with assistance of forceps and/or vacuum extraction and through CS respectively with males born vaginally without instrumental assistance and adjusted for: birthweights standardized according to gestational age gestational age,, maternal age, parity, maternal diseases (diabetes, hypertension, preeclampsia, SLE), highest parental educational level, household disposable income, parental country of birth and highest parental socioeconomic index.

[1]Model 1 compared all males in the main analytic sample, as well as those previously excluded because of extreme-value conscription data, born by CS to males born vaginally and adjusted identically to vaginal instrumental above.

[2]Model 2 compared all males in the main analytic sample born by CS to males born vaginally and adjusted as vaginal instrumental above with relaxed linearity assumptions, using cubic splines with five knots (5th, 27.5th, 50th, 72.5th and 95th percentiles), for maternal age, parental disposable income, gestational weight and birthweight standardized according to gestational age.

[3]Model 3 compared all males in the main analytic sample born by CS to males born vaginally and adjusted as vaginal instrumental above with additional adjustment for previous cesarean delivery.

[4]Model 4 compared all males born by CS 1982-87 in the analytic sub-population to males born vaginally and adjusted as vaginal instrumental above with additional adjustment for standardized maternal gestational weight gain, maternal smoking habits during pregnancy and maternal pre-pregnancy BMI.

[5]Model 5 compared all males in the main analytic sample born by CS to males born vaginally and adjusted as vaginal instrumental above with the subtraction of adjustment for gestational age.

[6]Model 6 compared all males in the main analytic sample, excluding all those born pre-term (<37 weeks), born by CS to males born vaginally and adjusted as vaginal instrumental above.

[7]Model 7 compared all males in the main analytic sample, including only those born at-term, born by CS to males born vaginally and adjusted as vaginal instrumental above.

Population characteristics of excluded cohorts

Supplementary Table S4

Outcome and Covariates	Among those who conscripted			
	**Main analytic sample (Males born 1972-87)** N=339,451	**No CRF test or extreme value[1]** N=274,038	**All conscripted[2]** N=613,489	**Not conscripted[2]** N=98,514
**Cesarean delivery, number (%)**	29,910 (8.8%)	30,090 (11.0%)	60,000 (9.8%)	11,926 (12.1%)
**Age at conscription (years), median (IQR)**	18.3 (18.1, 18.5)	18.3 (18.1, 18.6)	18.3 (18.1, 18.5)	N/A
**Birth weight (g), mean (SD)**	3,584.3 (530.9)	3,572.0 (541.5)	3,578.8 (535.7)	3,506.0 (600.6)
**Weeks of gestation (weeks), mean (SD)**	39.7 (1.8)	39.5 (1.8)	39.6 (1.8)	39.3 (2.1)
**Maternal age at birth (years), mean (SD)**	27.4 (4.9)	28.1 (5.1)	27.7 (5.0)	27.9 (5.2)
**Parity (number) median (IQR = Q3 – Q1)**	2.0 (1.0, 2.0)	2.0 (1.0, 2.0)	2.0 (1.0, 2.0)	2.0 (1.0, 2.0)
**Maternal diseases at time of giving birth**				
Diabetes, number (%)	1,233 (0.4%)	1,599 (0.6%)	2,832 (0.5%)	639 (0.6%)
Hypertension, number (%)	262 (0.1%)	477 (0.2%)	739 (0.1%)	188 (0.2%)
Preeclampsia, number (%)	1,459 (0.4%)	2,679 (1.0%)	4,138 (0.7%)	1,004 (1.0%)
Systemic lupus erythematosus, number (%)	29 (<1%)	29 (<1%)	58 (<1%)	12 (<1%)
**Highest parental educational level**				
Primary education, number (%)	43,816 (12.9%)	35,362 (12.9%)	79,178 (12.9%)	18,244 (18.5%)
Secondary education, number (%)	167,390 (49.3%)	140,158 (51.1%)	307,548 (50.1%)	50,520 (51.3%)
University degree or higher, number (%)	128,245 (37.8%)	98,518 (36.0%)	226,763 (37.0%)	29,750 (30.2%)
**Household disposable income (quintiles 1-5)**				
Quintile 1	27,715 (8.2%)	48,868 (17.8%)	76,583 (12.5%)	17,765 (18.0%)
Quintile 2	63,044 (18.6%)	61,105 (22.3%)	124,149 (20.2%)	21,187 (21.5%)
Quintile 3	85,334 (25.1%)	58,917 (21.5%)	144,251 (23.5%)	21,085 (21.4%)
Quintile 4	83,394 (24.6%)	54,769 (20.0%)	138,163 (22.5%)	19,916 (20.2%)
Quintile 5	79,964 (23.6%)	50,379 (18.4%)	130,343 (21.2%)	18,561 (18.8%)
**Parental country of birth**				
Both parents born in Sweden	299,243 (88.2%)	231,346 (84.4%)	530,589 (86.5%)	72,383 (73.5%)
One parent born in Sweden	29,445 (8.7%)	27,960 (10.2%)	57,405 (9.4%)	12,504 (12.7%)
Neither parent born in Sweden	10,763 (3.2%)	14,732 (5.4%)	25,495 (4.2%)	13,627 (13.8%)
**Highest parental occupational class in childhood**				
Self-employed, farmers and non-categorized	30,222 (8.9%)	30,283 (11.1%)	60,505 (9.9%)	12,695 (12.9%)
Unskilled workers	59,778 (17.6%)	48,776 (17.8%)	108,554 (17.7%)	21,461 (21.8%)
Skilled workers	65,380 (19.3%)	53,061 (19.4%)	118,441 (19.3%)	20,355 (20.7%)
Non-manual workers (lower level)	51,617 (15.2%)	40,170 (14.7%)	91,787 (15.0%)	12,824 (13.0%)
Non-manual workers (intermediate level)	86,444 (25.5%)	61,454 (22.4%)	147,898 (24.1%)	19,123 (19.4%)
Non-manual workers (higher level)	46,010 (13.6%)	40,294 (14.7%)	86,304 (14.1%)	12,056 (12.2%)

[1]Individuals who were eligible for conscription but who were not considered medically fit during conscription testing due to injuries, abnormal resting ECG or other medically relevant indications were not invited to perform the maximum load adjustable resistance cycle ergometer test.

[2] During the period of study, conscription was mandatory by law and exemption from conscription required stated approval, often granted for those with severe medical conditions
